# Plutonium Signatures in a Dated Sediment Core as a Tool to Reveal Nuclear Sources in the Baltic Sea

**DOI:** 10.1021/acs.est.2c07437

**Published:** 2023-01-23

**Authors:** Mercedes López-Lora, Grzegorz Olszewski, Elena Chamizo, Per Törnquist, Håkan Pettersson, Mats Eriksson

**Affiliations:** † Department of Health, Medicine and Caring Sciences (HMV), Linköping University, 58183Linköping, Sweden; ‡ Faculty of Chemistry, Department of Environmental Chemistry and Radiochemistry, Laboratory of Toxicology and Radiation Protection, University of Gdańsk, Wita Stwosza 63, 80-308Gdańsk, Poland; § Centro Nacional de Aceleradores (CNA), Universidad de Sevilla, Junta de Andalucía, Consejo Superior de Investigaciones Científicas, Parque científico y tecnológico Cartuja, Thomas Alva Edison 7, 41092Sevilla, Spain; ∥ Department of Medical Radiation Physics, and Department of Health, Medicine and Caring Sciences, Linköping University, 58183Linköping, Sweden

**Keywords:** Pu isotopes, plutonium-244, Baltic Sea, sediment, global fallout, Studsvik nuclear facility, radioactive liquid discharges, accelerator mass spectrometry

## Abstract

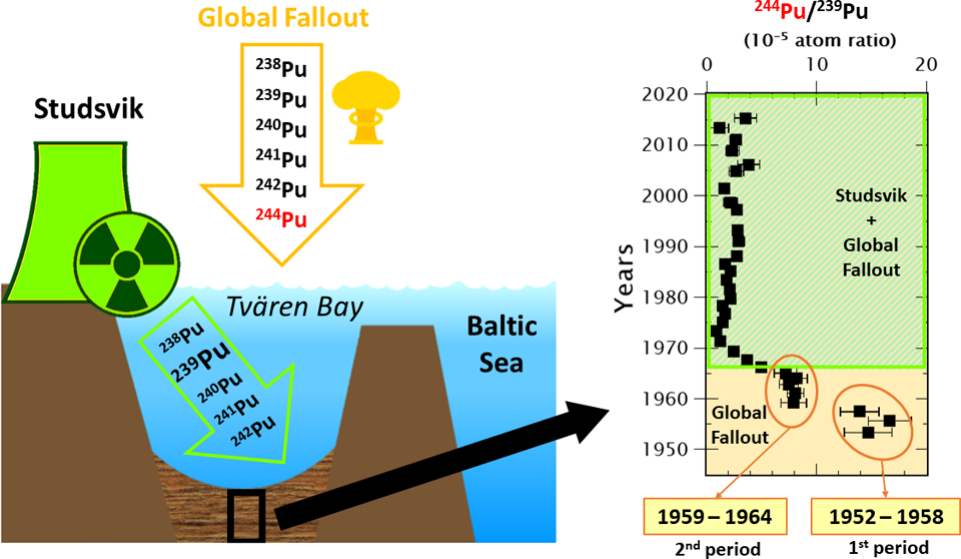

Plutonium distribution was studied in an undisturbed sediment core sampled from the Tvären bay in the vicinity of the Studsvik nuclear facility in Sweden. The complete analysis, including minor isotopes, of the Pu isotope composition (^238^Pu, ^239^Pu, ^240^Pu, ^241^Pu, ^242^Pu, and ^244^Pu) allowed us to establish the Pu origin in this area of the Baltic Sea and to reconstruct the Studsvik aquatic release history. The results show highly enriched ^239^Pu, probably originating from the Swedish nuclear program in the 1960s and 1970s and the handling of high burn-up nuclear fuel in the later years. In addition, the ^244^Pu/^239^Pu atomic ratio for the global fallout period between 1958 and 1965 is suggested to be (7.94 ± 0.31)·10^–5^. In the bottom layer of the sediment, dated 1953–1957, we detected a higher average ^244^Pu/^239^Pu ratio of (1.51 ± 0.11)·10^–4^, indicating the possible impact of the first US thermonuclear tests (1952–1958).

## Abstract 2

This work reveals unknown Pu discharges to the Baltic Sea from the Studsvik Nuclear facility (Sweden) by analyzing a sediment core. The ^244^Pu/^239^Pu ratio is used to unravel the global fallout signal.

## 1 Introduction

In the years following World War II, many countries considered developing nuclear energy for power and weapons production. Sweden was no exception, and the Swedish nuclear program was initiated in 1945. In the beginning, Sweden was working with the idea of using heavy water reactors to produce nuclear power and Pu material for weapons. The nuclear program was first controlled by one state-owned institution but later divided into two major organizations, the Swedish National Defense Research Institute (FOA) and AB Atomenergi (AE). In 1959, AE started a large-scale nuclear research institute about 100 km South of Stockholm, later in this paper referred to as Studsvik. Different research programs were conducted within this facility, including work on Pu separation and irradiated fuel. The Swedish nuclear weapon program, undertaken mainly by FOA, was decommissioned in 1972 when Sweden joined the Non-Proliferation Treaty (NPT). Equipment from the FOA facility was later transported to Studsvik for decontamination and prepared for final storage.^1^

**Figure 1:**
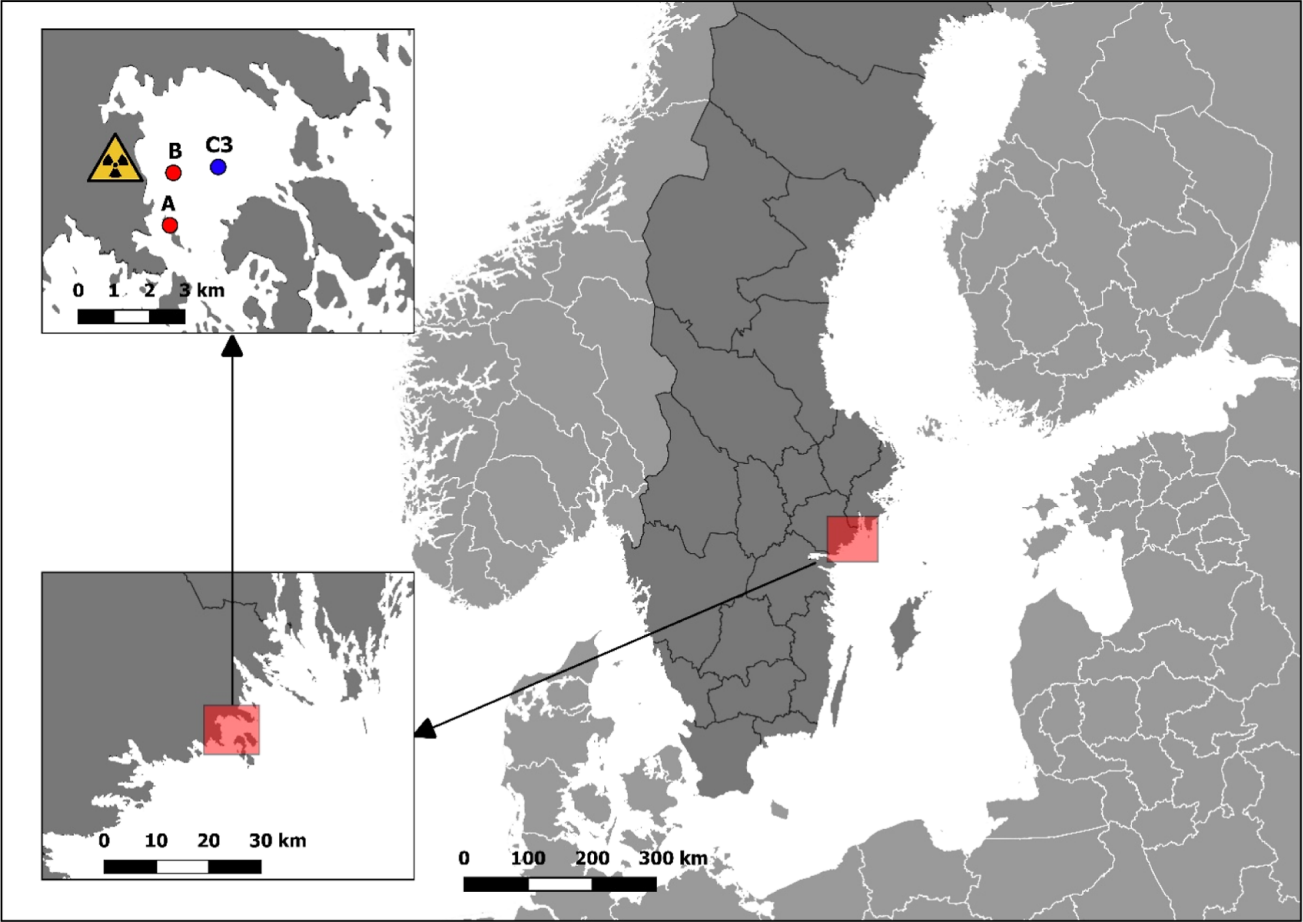
Tvären location on the Swedish coast of the Baltic Sea with the marked Studsvik nuclear research facility, the sediment sampling station, core C3 (blue dot), and discharge points (red dots).

The Studsvik nuclear research facility is situated at the Swedish coast of the Baltic Sea, on the shore of the Tvären bay (Figure 1). Due to the unique characteristics of the Tvären bay (shallow inlets and deep central area) and periodic anoxic conditions at the bottom, sedimentation processes of different radionuclides can be studied. Today, Studsvik mainly processes radioactive wastes, resulting in periodic liquid discharges containing various radionuclides.^2,3^ The Swedish Radiation Safety Authority (SSM) regulates permissible levels of these aquatic releases. Liquid discharges have been documented since the beginning of the operations in 1959, although the composition of the releases had not been well described before the 1970s. Some gamma emitters and total alpha activities have been reported since the operation’s beginning, and since 2002, the reported radionuclides are extended to include, for example, ^238^Pu and ^239+240^Pu. However, the release data do not include the minor Pu isotopes like ^241,242,244^Pu or separate information on ^239^Pu and ^240^Pu. Nowadays, there are two operational discharge points with regular higher radioactive releases in the southern part of the Tvären bay and lower radioactive releases close to the Studsvik facility (Figure 1).

Tvären, as a part of the Baltic Sea, has received Pu from other sources than Studsvik. Naturally occurring Pu can be considered negligible in the general environment.^4^ However, multiple nuclear activities have spread this element worldwide since the beginning of the nuclear age in July 1945, when the first nuclear bomb was detonated at the Trinity test site (New Mexico, USA). Atmospheric nuclear weapon tests were conducted between 1945 and 1980. Radionuclide fallout from these tests constitutes a global input that has covered the Earth’s surface, also called global fallout (GF). The GF peaked around in 1963 with maximum deposition in the Northern Hemisphere (30–70° N) and is considered the main Pu input to the general environment.^5^ In addition, there are other sources with a local/regional impact, for example, control releases from nuclear reprocessing facilities (the most important ones in Europe are Sellafield, United Kingdom, and La Hague, France) and fallout coming from the Chernobyl accident in 1986.^6,7^ The Pu isotopic composition varies according to the production mode, providing a unique “fingerprint” of different sources and becoming a key tool for many environmental applications (Table 1). The most frequently studied Pu isotopes in the environment have been the alpha-emitters ^238^Pu (*t*_1/2_ = 87.7 y), ^239^Pu (*t*_1/2_ = 2.411 × 10^4^ y), and ^240^Pu (*t*_1/2_ = 6.561 × 10^3^ y), where ^239^Pu and ^240^Pu often are given as combined alpha activities, that is, ^239+240^Pu. However, more scarce data are available for the presence of minor Pu isotopes in the environment, that is, ^241^Pu (*t*_1/2_ = 14.2*y*), ^242^Pu (*t*_1/2_ = 3.75 × 10^5^*y*), and ^244^Pu (*t*_1/2_ = 8.13 × 10^7^ y). Thus, open questions about the origin of Pu could be solved by studying the complete Pu isotopic composition.

**Table 1:** Activity and Atomic Ratios for Pu from Diverse Sources

source	^238^Pu/^239+240^Pu (in 2016) activity ratio	^240^Pu/^239^Pu atom ratio	^241^Pu/^239^Pu (in 2016) atom ratio	^242^Pu/^239^Pu atom ratio	^244^Pu/^239^Pu atom ratio
GF NH (1945–1980)	0.022^7^	0.180 ± 0.014^5^; 0.1808 ± 0.0057^40^	(8.6 ± 1.2)·(10^–4^)^5^;(7.2 ± 0.5)·(10^–4^)^41^	(3.87 ± 0.71)·(10^–3^)^5^; (4.0 ± 0.3)·(10^–^^3^)^41^	(5.7 ± 1.0)·(10^–5^)^9^
GF + SNAP 9A NH	0.022–0.034^7^; 0.039 ± 0.014^42^				
Chernobyl	0.31–0.42^7, 43^	0.408 ± 0.003^44^	0.028–0.033^44,45^	0.043^40,44,45^	
weapon-grade Pu	0.0161 ± 0.0005^46^; 0.009–0.023 (Thule)^47^	0.01–0.07^48^; 0.0551 ± 0.0008 (Thule)^46^; 0.023–0.054 (Thule)^49^; 0.0564–0.0636 (Marshall Islands)^50^	8–12·(10^–5^) (Nagasaki)^51^; 1–7.2·(10^–5^) (Montebello)^51^; < 2.3·(10^–4^)^11^	2.5–4.3·(10^–4^)^11,52^	
MOX fuel	0.56–8.15^53,54^	0.27–0.78^53,54^	0.06–0.32^54, 55^	0.01–0.21^53,54^	
nuclear fuel reprocessing plants	0.19 (Sellafield)^55^; 0.36 (Cap La Hague)^55^	0.16–0.24 (Sellafield)^56^; 0.34 ± 0.03 (Cap La Hague)^55^	(8.45 ± 0.12)·(10^–3^) (Sellafield)^9^	(6.4 ± 0.7)·(10^–3^) (Sellafield)^57^	<3.5·10^–6^ (Sellafield)^9^
Fukushima	1.01–2.80^58^	0.32–0.33^58^	0.105–0.111^58^		
Enewetak Atoll, Ivy Mike (1952)	<0.01^59^	0.363 ± 0.004^10^	(1.79 ± 0.02)·(10^–3^)^10^	0.019 ± 0.003^10^	(1.18 ± 0.07)·(10^–^^3^)^10^
Bikini Atoll, Operation Castle (1954)	0.001^60^	0.276 ± 0.011^61^; 0.263 ± 0.003^15^	(1.7 ± 0.3)·(10^–3^)^15^		(2.5 ± 0.4)·(10^–^^4^)^15^
Bikini Atoll soils and sediments		0.288–0.323^15^	1.4–3.8·(10^–3^)^15^		3.1–5.7·(10^–^^4^)^15^

In the last decade, some studies have pointed out the interest in one of the most understudied Pu isotopes, ^244^Pu. Although this radionuclide has been primarily known for its applications in astrophysics,^12−14^ anthropogenic ^244^Pu could be an innovative tool due to the recently developed techniques to assess this radionuclide at environmental levels. ^244^Pu can only be produced in thermonuclear explosions, where the fast neutron flux triggers its production after successive neutron capture reactions on ^239^Pu.^10,15^ Such processes are very unlikely in civil nuclear reactors due to the low neutron flux and the production of the short-lived ^243^Pu (*t*_1/2_ = 4.956 h) by neutron capture of ^242^Pu, which decays in the reactor before ^244^Pu is produced.^9,16^ This unique aspect of ^244^Pu makes it ideal to differentiate Pu originating from thermonuclear explosions and other Pu sources, for example, fission weapon fallout and discharges from the nuclear fuel cycle. Several studies have been reported^9,11,15−17^ establishing the first ^244^Pu/^239^Pu ratios for different sources (Table 1). Still, a complete study including ^244^Pu to correctly identify the GF Pu contribution in the context of multiple sources has not been reported.

In this work, we used an undisturbed and ^210^Pb-dated sediment core to investigate the complete Pu isotopic composition for Pu source identification in the vicinity of the Studsvik nuclear facility (Figure 1). We used the ^244^Pu/^239^Pu atom ratio to unravel the GF contribution and to reconstruct the earlier unknown Pu releases from Studsvik. The results revealed the past and present Pu-related activities conducted by Studsvik. The obtained ^244^Pu/^239^Pu atomic ratios in the deepest sediment layers were used to confirm the possible presence of Pu originating from early thermonuclear tests performed by the USA in the Marshall Islands (Pacific Ocean, 1952–1958).

## 2 Materials and Methods

### 2.1 Samples

A 46-cm-long sediment core, C3, was sampled in the deepest part (79 m) of the Tvären bay (Figure 1) on the 13th of October, 2016, using a gravity corer. The bottom water was anoxic at the site, and no signs of bioturbation could be seen. The liquid discharges from Studsvik are released in the Tvären bay via two pipelines (see the new Figure 1, point A at 10 m and point B at 6 m). Pipeline A is the main release point. Discharges from pipeline B are regulated to a maximum of one-tenth of the total discharged activities from the Studsvik facility. The chosen sampling station is assumed to be the most representative place for both discharge points. Following sediment sampling, the core was promptly cut into 1 cm slices ashore and then transported to the laboratory where sediment slices were freeze-dried. Around 4 g of subsamples was taken from each core slice and prepared for the radiochemical separation of plutonium. The constant rate of supply (CRS) method was used for sediment dating after measuring ^210^Pb (^210^Po). The model was fine-tuned by using a clearly defined time marker in the sediment profile, that is, stable Pb concentration that had a maximum in 1970, and the results have been validated by using additional time markers according to the Studsvik discharge history records for ^60^Co and ^152^Eu (Supporting Information, Figure S3). These data will be published elsewhere.^18^

### 2.2 Radiochemical Separation of Pu

Before analysis, all subsamples were again dried overnight at 80 °C to ensure that possible adsorbed water during storage was removed. Sediment subsamples were calcined at a low temperature, 450 °C, to prevent the formation of refractory Pu species.^19^ Two groups of samples were processed: (i) the first set of samples (group 1) was intended for alpha spectrometry analysis and they were initially spiked with ^242^Pu to quantify the final Pu concentrations, and (ii) the second set of samples (group 2) were processed to study the ^240^Pu/^239^Pu, ^241^Pu/^239^Pu, ^242^Pu/^239^Pu, and ^244^Pu/^239^Pu atom ratios by accelerator mass spectrometry (AMS) and no ^242^Pu spike was added. Incinerated sediment samples were leached for 2 to 3 days using aqua regia, and actinides were coprecipitated with Fe(OH)_3_ from the dissolved fraction. The Fe(OH)_3_ precipitate was dissolved in concentrated HCl and diluted with MQ H_2_O, and K_2_S_2_O_5_ was added to reduce Pu to Pu(III). Additionally, ascorbic acid was added to assure complete Fe reduction. Reduced forms of Pu were coprecipitated on Fe(OH)_2_, dried, and dissolved in 8 M HNO_3_. The separation was performed on TEVA resin cartridges coupled to a vacuum box. The cartridge was rinsed with 8 M HNO_3_ and 1 M HNO_3_ (Am and U elution) and 9 M HCl (Th elution). The Pu fraction was eluted using 2 M HCl/0.1 M NH_2_OH·HCl and, after decomposition, prepared for electrodeposition on stainless-steel discs from Na_2_SO_4_/H_2_SO_4_ solution adjusted to pH 2.1–2.4 with NH_3_·H_2_O.^20^ Finally, after alpha spectrometric measurements, samples were adapted for their analysis by AMS. Discs were leached in 3 M HNO_3_, and Pu was coprecipitated as Fe(OH)_3_ after adding 1 mg of Fe(III). Precipitates were dried and incinerated at 650 °C, mixed with 3 mg of Nb powder, and pressed into aluminum cathodes.^21^ See Section S1 from the Supporting Information for more details.

### 2.3 Measurements

The Pu discs were stored for 2 weeks before the alpha spectrometric analysis to enable corrections for any remaining ^228^Th in the ^238^Pu region of interest using the ^224^Ra alpha peak for calculating ^228^Th. ^238^Pu and ^239+240^Pu measurements were performed on a 1024-channel Alpha Analyst Canberra alpha spectrometer. The discs were measured between 15 and 33 days. MDA (i.e., minimum detectable activity) values were 0.09 mBq·g^–1^ for ^239+240^Pu and 0.08 mBq·g^–1^ for ^238^Pu for 2 weeks measurements calculated using the revised Currie formula.^22^ The QA/QC of the method was controlled using the IAEA-135 reference material. See Section S1 from the Supporting Information for more details.

^240^Pu/^239^Pu, ^241^Pu/^239^Pu, ^242^Pu/^239^Pu’, and ^244^Pu/^239^Pu isotopic ratio determinations were carried out using the compact 1 MV AMS system at the CNA (Centro Nacional de Aceleradores, Sevilla, Spain), following the technique for Pu measurements described in previous studies.^21,23^ For the control of the measurement stability and the necessary standard correction of the isotopic ratios, the ColPuS standard^24^ was also analyzed. Furthermore, instrumental (i.e., untreated iron oxide matrixed mixed with Nb) and procedural blanks were included in the measurement sequence to control the background from the AMS measurement and from the laboratory (i.e., multiple procedural blanks were prepared according to the different sample groups processed, see Section S1 of the Supporting Information for details). An additional correction was included in the case of ^241^Pu to subtract the in-grown ^241^Am on the overall detected 241 mass counts. For this, the elapsed time between the sample preparation and the AMS analysis was taken into account and a 10% higher production of AmO^–^ ions was considered in the Cs sputter ion source compared to that of PuO^–^.^21,25^ Thus, between 0.5 and 16% of the overall detected 241 mass counts were subtracted following this proxy (i.e., 5–16% in the samples from group 1 and <1% in samples from group 2, see Section S1 from the Supporting Information).

## 3 Results

### 3.1 Plutonium Concentrations and Atomic Ratios

**Figure 2:**
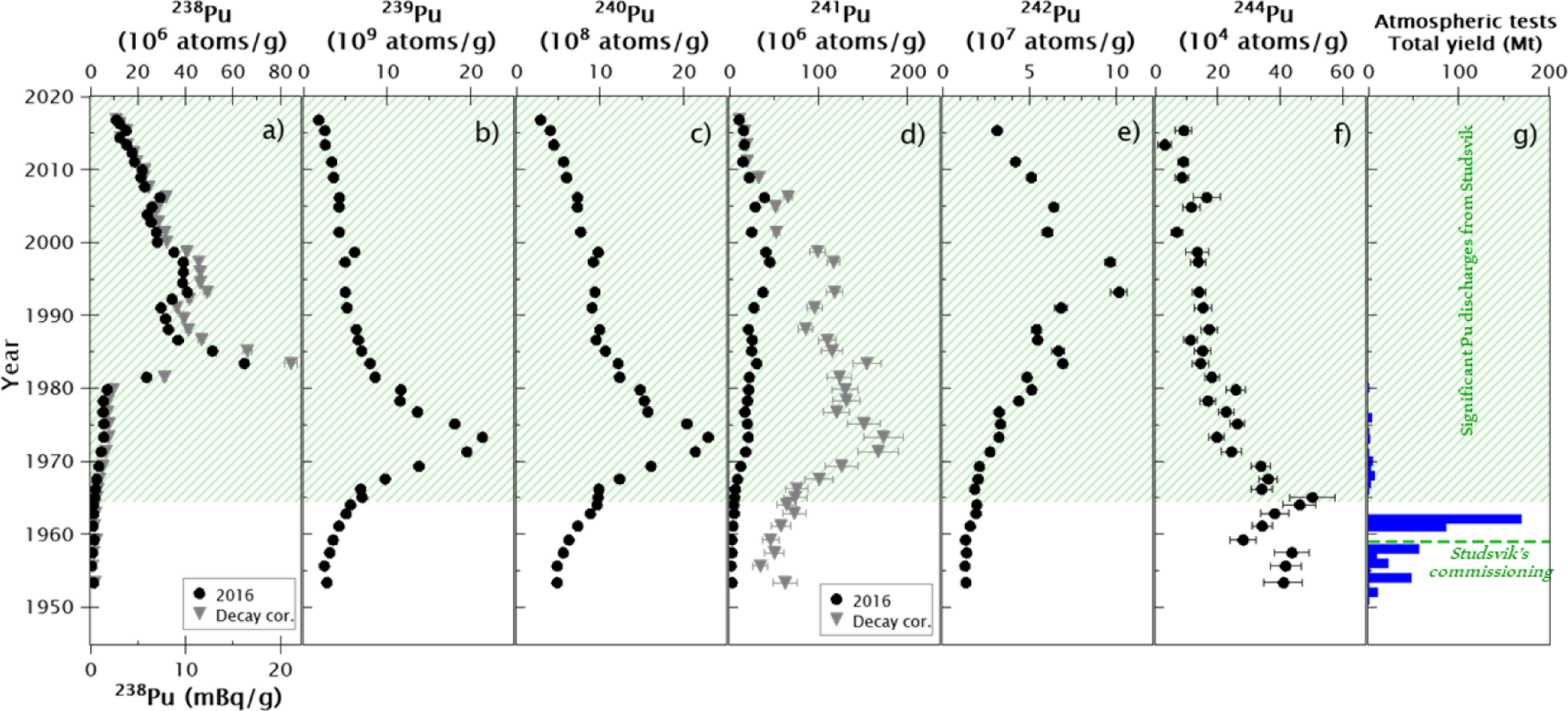
Depth distribution of Pu isotopes in the sediment core C3. ^238^Pu and ^241^Pu concentrations are decay corrected to 2016 (black dots) and to the date of the layer according to the ^210^Pb dating method (gray triangles). Plot (g) shows the total yield from the atmospheric nuclear tests (1945–1980),^27^ and the green dashed line indicates the commission year of the Studsvik nuclear research facility. The green dashed area represents the layers of the sediment core significantly affected by the Pu discharges from Studsvik (Section 4.1). The uncertainties of the date of the layers according to the ^210^Pb dating method are detailed in Figure S3.

**Figure 3:**
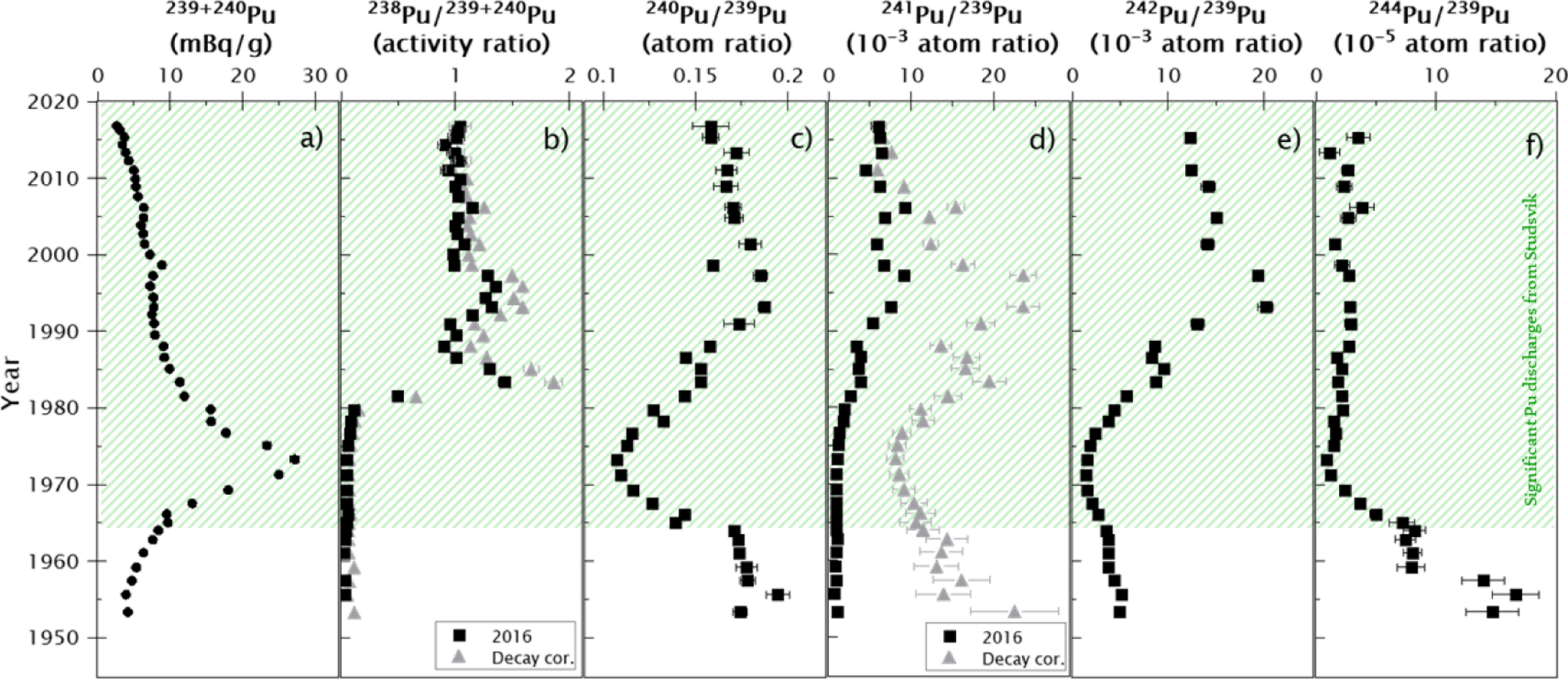
Measurement results from the C3 sediment core. ^239+240^Pu activity concentrations left graph (black dots) and the different Pu isotopic ratios (black squares) from core C3. ^238^Pu and ^241^Pu ratios are decay corrected to 2016 (black squares) and the date of the layer according to the ^210^Pb dating method (gray triangles). The green dashed area represents the layers of the sediment core significantly affected by the Pu discharges from Studsvik (Section 4.1). The uncertainties of the date of the layers according to the ^210^Pb dating method are detailed in Figure S3.

Concentrations depth profiles of ^238^Pu (α-spectrometry) and ^239^Pu, ^240^Pu, ^241^Pu, ^242^Pu, and ^244^Pu (AMS) for the studied sediment core are given in Figure 2. Since ^238^Pu and ^241^Pu have partially decayed since their initial deposition, two depth profiles are shown in those cases: one decay corrected to the sampling date (2016-10-13) and one decay corrected to the date of the layer according to the ^210^Pb dating method. Additionally, the overall detonated yield during the atmospheric testing period (1945–1980) is shown to compare the plutonium isotope concentrations with the intensity of the historical nuclear tests. The ^238^Pu/^239+240^Pu activity ratios and ^240^Pu/^239^Pu, ^241^Pu/^239^Pu, ^242^Pu/^239^Pu, and ^244^Pu/^239^Pu atomic ratios are shown in Figure 3. All the results are given with the calculated combined standard uncertainty. Duplicate samples were analyzed, proving the reliability of these results (Supporting Information, Section S1 and Figure S1).

The Pu activity inventory at the sampling location has been estimated to be (383.5 ± 4.3) Bq/m^2^ and (802.5 ± 8.1) Bq/m^2^ for ^238^Pu and ^239+240^Pu, respectively. These values are significantly higher than the previously reported inventories from different stations along the Baltic Sea, ranging from (63 ± 1) to (159 ± 3) Bq/m^2^ for ^239+240^Pu and from (2.8 ± 0.1) to (7.8 ± 0.3) Bq/m^2^ for ^238^Pu.^26^ For the remaining Pu isotopes measured by AMS, atomic inventories of (579.7 ± 6.4)·10^12^ atoms/m^2^ for ^239^Pu, (83.18 ± 0.91)·10^12^ atoms/m^2^ for ^240^Pu, (1.685 ± 0.026)·10^12^ atoms/m^2^ for ^241^Pu, and (1.793 ± 0.051)·10^10^ atoms/m^2^ for ^244^Pu have been obtained.

## 4 Discussion

### 4.1 Unraveling the GF Pu Signature Using ^244^Pu

Pu discharges from the Studsvik nuclear research facility started in 1959, which coincides with the most intensive nuclear test period (Figure 2g). Due to that, the GF Pu signal is masked and challenging to unravel. Since ^244^Pu is produced only in the thermonuclear explosions, no ^244^Pu is expected to be present in the Studsvik discharges or in any other sources in the vicinity of the sampling site, ^244^Pu results are suitable to calculate the GF Pu fraction.

The atmospheric nuclear testing can be divided into two periods according to the fission yield and the number of explosions for the most powerful tests (>4 Mt). Thus, the earlier thermonuclear weapons testing phase (1952–1958) was dominated by the U.S. program (from now on the first GF period), whereas the later years up the Treaty Banning Nuclear Weapon Tests in the Atmosphere in 1963 (i.e., from now on the second GF period) were mostly dominated by the USSR tests, with the global deposition peaking in 1963.^27^

**Figure 4:**
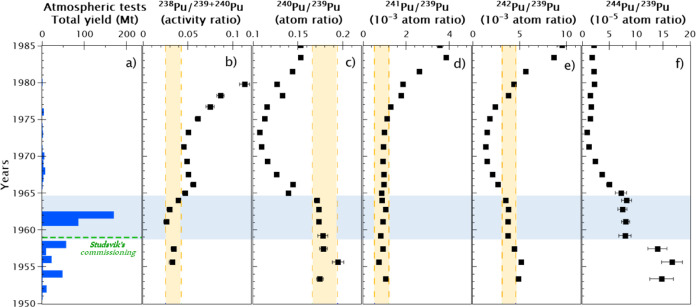
^244^Pu depth distribution and Pu isotopic ratios in the sediment core C3 from 1950 to 1985. ^238^Pu/^239+240^Pu and ^241^Pu/^239^Pu ratios are decay corrected to 2016 (i.e., sampling date). The left plot (a) represents the total yield from the atmospheric nuclear test^27^ (1945–1980), and the green dashed line indicates the commission year of the Studsvik nuclear research facility. The blue area highlights the period from 1959 to 1964, where all the Pu isotopic ratios are as expected from the GF (second period, see the discussion in Section 4.1). The uncertainties of the date of the layers according to the ^210^Pb dating method are detailed in Figure S3.

^244^Pu concentrations in the sediment core agree with this pattern (Figure 2f). A ^244^Pu peak is observed in the sediment deposited between 1963 and 1966, followed by a decreasing trend from this point to the upper younger sediment layers. This distribution pattern is similar to the expected pattern dominated by the main GF input in 1963 after the second GF testing period, as observed in other studies.^28,29^ Additionally, an increase in the ^244^Pu concentrations is observed in the three deepest samples, which might be a signal of the first GF testing period, as is discussed in the next section. This GF peak in 1963–1966 is not observable for other Pu isotopes as these isotopes are more pronounced in the Studsvik discharges. In the years after 1965, a clear increase in concentrations for all the Pu isotopes (except for ^244^Pu) is observed (Figure S2). Knowing the Pu isotopic ratios, it is possible to identify the sediment layers that are only affected by the GF. Obvious deviations from the expected Pu characteristic values from the GF (Table 1) are observed along the sediment core; however, although Studsvik commissioning was in 1959, no significant Pu signal from Studsvik is present in sediment layers formed earlier than 1964 (Figure 4). Therefore, samples from the bottom of the sediment core dated up to 1958 are considered representative of the first GF period and they are discussed in the next section. Likewise, samples in the sediment layers dated between the years 1959 and 1964 are considered representative of the second GF period, with an average activity ratio of 0.0393 ± 0.0033 for ^238^Pu/^239+240^Pu and average atom ratios of 0.1738 ± 0.0027, (9.1 ± 1.0)·10^–4^, and (3.70 ± 0.13)·10^–3^ for ^240^Pu/^239^Pu, ^241^Pu/^239^Pu, and ^242^Pu/^239^Pu, respectively. These ratios are in good agreement with the previously reported characteristic integrated GF ratios (Table 2), so this second GF period is expected to be the dominant one up to now. Therefore, we can assume that the characteristic GF signature (Pu isotopic ratios) has not changed since 1964, as has been previously observed in other studies.^30^ Likewise, the mean ^244^Pu/^239^Pu atom ratio of (7.94 ± 0.31)·10^–5^ derived from these sediment layers can be considered representative of the GF ^244^Pu/^239^Pu ratio in the second GF period. In a previous work,^9^^244^Pu/^239^Pu ratios were reported from two soil samples collected at Salzburg (Austria), only affected by GF. The reported values of (6.35 ± 0.11)·10^–5^ and (5.09 ± 0.16)·10^–5^ are in reasonable agreement with our GF value considering the scarcity of the results and being integrated values for soil samples from a different latitude. To the best of our knowledge, these are the only reported values regarding the ^244^Pu/^239^Pu signal from GF.^9^ For further discussion, our derived ^244^Pu/^239^Pu characteristic atom ratio, that is, (7.94 ± 0.31)·10^–5^, is considered the most representative for the second GF period and from our sediment core since the layer dated 1959 to the top layer.

**Table 2:** Average Pu Isotopic Ratios from the Sediment Layers of the Sediment Core Corresponding to the First (1952–1958) and the Second (1959–1964) Nuclear Testing Periods^a^

	^238^Pu/^239+240^Pu activity ratio	^240^Pu/^239^Pu atom ratio	^241^Pu/^239^Pu (10^–4^) atom ratio	^242^Pu/^239^Pu (10^–3^) atom ratio	^244^Pu/^239^Pu (10^–5^) atom ratio
this work (1952–1958)	0.0379 ± 0.0055	0.1806 ± 0.0057	8.94 ± 0.93	4.81 ± 0.20	15.1 ± 1.1
this work (1959–1964)	0.0393 ± 0.0033	0.1738 ± 0.0027	9.1 ± 1.0	3.70 ± 0.13	7.94 ± 0.31
this work (1952–1964)	0.0386 ± 0.0041	0.1775 ± 0.0079	9.0 ± 1.2	4.17 ± 0.64	11.0 ± 3.9
bibliography (1952–1964)	0.022^7^	0.180 ± 0.014^5^	8.6 ± 1.2^5^	3.87 ± 0.71^5^	5.7 ± 1.0*^9^

a The average Pu ratios for the complete nuclear atmospheric testing are also calculated and compared to the reported GF ratios in the previous studies, that is integrated for the whole nuclear atmospheric testing. *Average ratio from two soil samples collected from Austria.^9^

**Figure 5:**
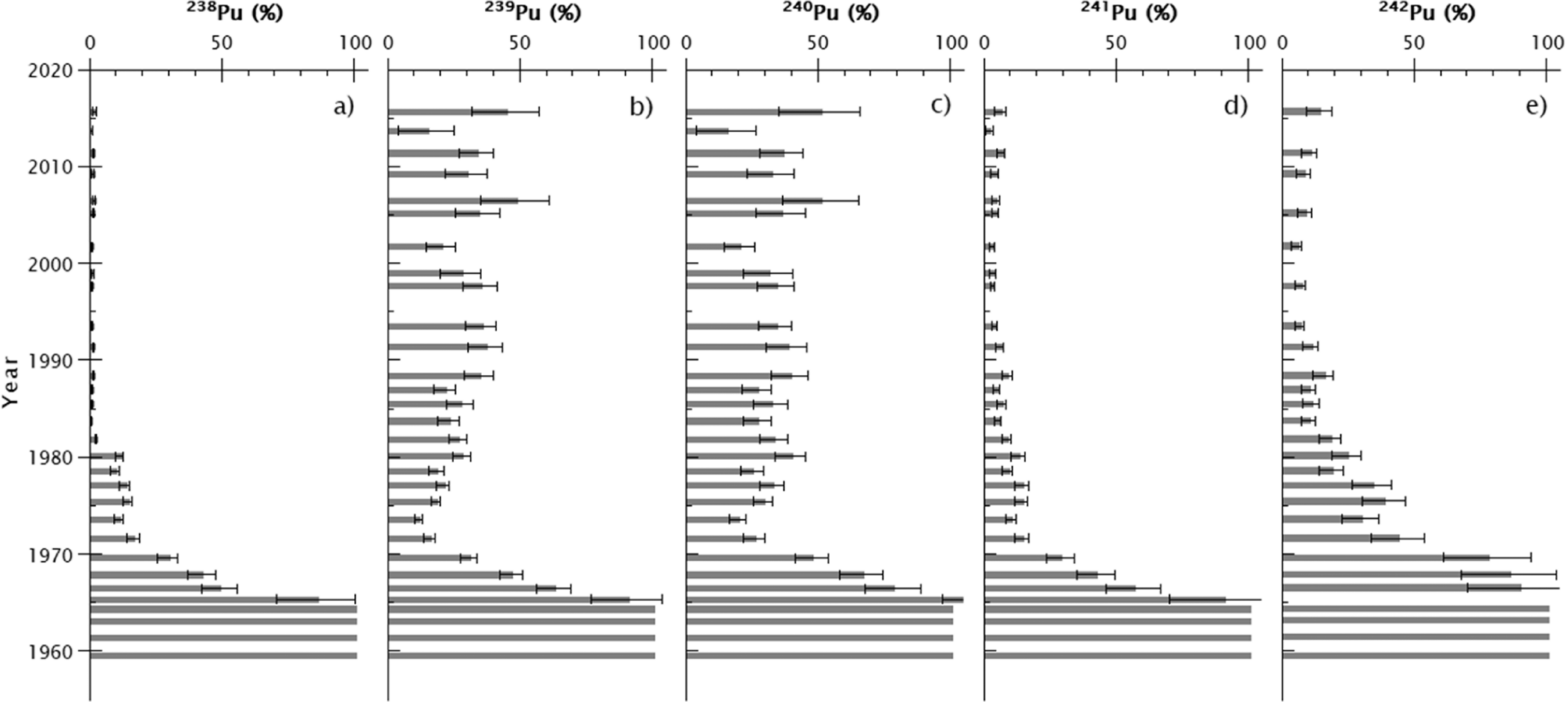
Fraction of Pu originating from the GF in the sediment core. For sediment layers deposited earlier than 1965, 100% of the Pu is considered to originate from the GF. The uncertainties of the date of the layers according to the ^210^Pb dating method are detailed in Figure S3.

From the ^244^Pu/^239^Pu results, the contribution of ^239^Pu from GF in the sampled core can be calculated using a two-end-member linear mixing model, as previously used for the other Pu isotopic ratios.^31^ Assuming only two sources, that is, GF and Studsvik, and considering that no ^244^Pu released from Studsvik, the model is simplified, and the ^239^Pu GF fraction is given by
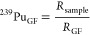
1where *R*_Sample_ is the measured ^244^Pu/^239^Pu atom ratio in the sample, *R*_GF_ is the ^244^Pu/^239^Pu characteristic ratio for GF, that is, (7.94 ± 0.31)·10^–5^, and ^239^Pu_GF_ is the fraction of ^239^Pu originating from GF. The corresponding GF fractions for other Pu isotopes (^*x*^Pu_GF_, *x* = mass numbers 238, 240, 241, and 242) can be calculated using the ^239^Pu_GF_ fraction and the characteristic Pu isotopic ratios for the GF (Table 2). The results for the GF fraction of the different Pu isotopes from all the analyzed sediment layers are shown in Figure 5. Note that 100% contribution from GF is assumed in sediment layers deposited earlier than or equal to 1964, as discussed above. Similarly, from the total Pu isotope inventory in the core, GF contributions of (3 ± 1), (39 ± 2), (49 ± 4), (12 ± 2), and (22 ± 4)% for ^238^Pu, ^239^Pu, ^240^Pu, ^241^Pu, and ^242^Pu, respectively, have been estimated. This means a total inventory of 342 ± 16 Bq/m^2^ for ^239+240^Pu from the GF at the sampling location, which is almost 1 order of magnitude higher than the average GF deposition in the Baltic Sea,^7^ that is, 40–50 Bq/m^2^. Additionally, these GF contributions give evidence of a high impact from Studsvik aquatic discharges at the sampling site, significant for all the studied Pu isotopes (except for ^244^Pu as previously discussed), with a total contribution ranging from about 50% in the case of ^240^Pu to about 97% for ^238^Pu.

### 4.2 Signal from the Earliest Thermonuclear Tests

**Figure 6:**
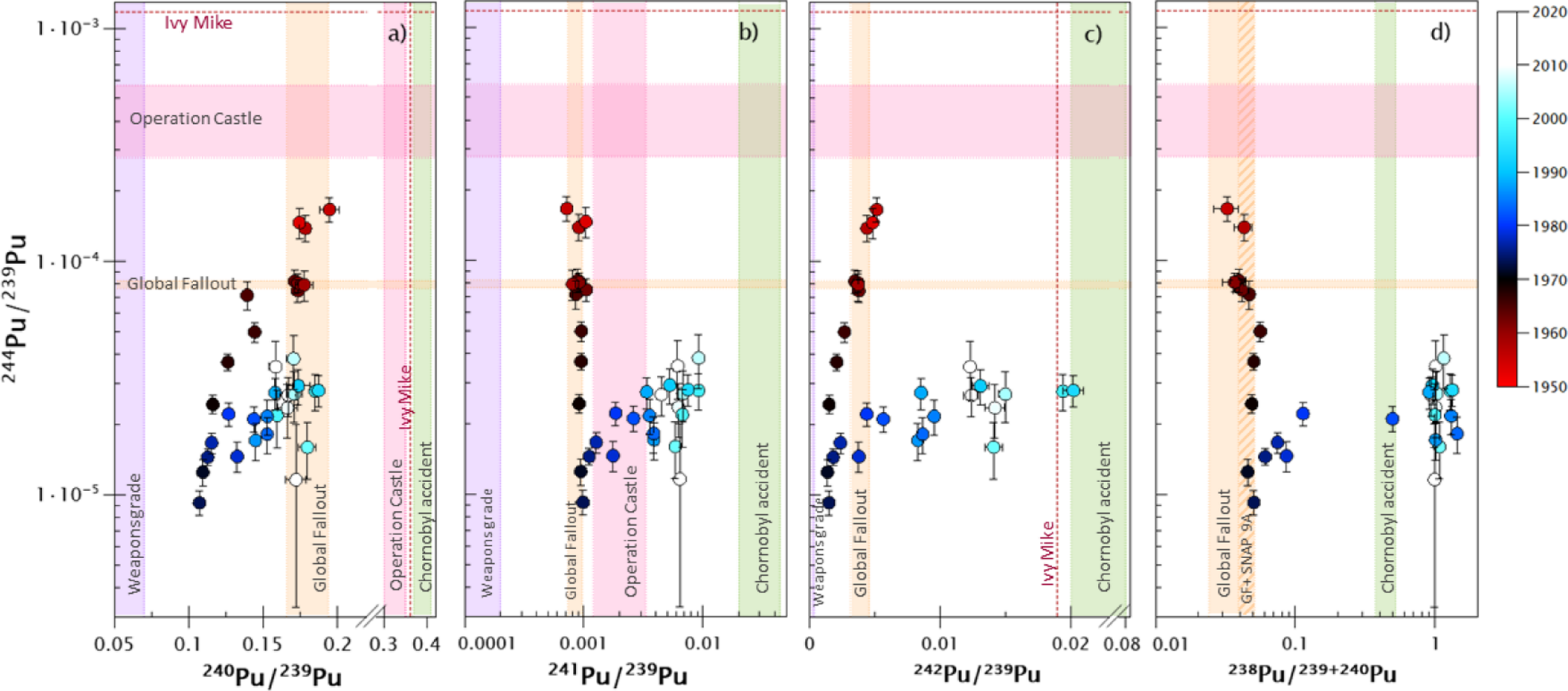
Source identification in different sediment layers using characteristic ^244^Pu/^239^Pu ratios vs ^240^Pu/^239^Pu, ^241^Pu/^239^Pu, ^242^Pu/^239^Pu, and ^238^Pu/^239+240^Pu. Pu isotopic ratios from the sediment core C3 and reported ratios from known Pu sources in the environment (Table 1). The GF ^244^Pu/^239^Pu ratio is taken from this work as discussed in Section 4.2. All the ratios are shown as atom ratios except for the ^238^Pu/^239+240^Pu activity ratio. ^241^Pu/^239^Pu and ^238^Pu/^239+240^Pu values are decay corrected to 2016. The year when the sediment layers were formed (dated using ^210^Pb dating) is color-coded in the graphs. The uncertainties of the date of the layers according to the ^210^Pb dating method are detailed in Figure S3.

Figure 4f shows a clear increment in the ^244^Pu/^239^Pu atomic ratios in the three deepest sediment layers dated between 1953 and 1957. Remarkably, high ^244^Pu/^239^Pu atom ratios (1.51 ± 0.11)·10^–4^ were measured during this period, clearly deviating from the measured ratio of (7.94 ± 0.31)·10^–5^ observed in sediment layers deposited between 1959 and 1964, that is, when GF showed a maximum deposition, as discussed in Section 4.2. Moreover, ^244^Pu concentration is characterized by a different depth distribution from that of the other Pu isotopes in layers dated from 1953 to 1958 (Figures 2 and S2), which suggests an enhanced production of ^244^Pu during this nuclear test period. Figure 6 presents the correlation between ^244^Pu/^239^Pu and the other Pu atom ratios with a color code indicating the sediment layer’s computed age. In addition, we have superimposed characteristic atom ratios reported for other Pu sources (Table 1) in the graphs for comparison purposes. The ^238^Pu/^239+240^Pu activity ratios and the ^240^Pu/^239^Pu and ^241^Pu/^239^Pu atom ratios measured in the three oldest sediment layers agree well with the characteristic GF Pu ratios. Additional information is provided by the ^244^Pu/^239^Pu and ^242^Pu/^239^Pu atomic ratios. Thus, the ^242^Pu/^239^Pu average isotopic ratio from these three samples (i.e., weighted average considering the uncertainties) is (4.81 ± 0.20)·10^–3^, a value slightly above the average GF ratio computed for sediment layers dated 1959–1964 [i.e., (3.70 ± 0.13)·10^–3^]. This different pattern is confirmed by the ^244^Pu/^239^Pu isotopic ratios, providing a more substantial indication of the influence of the thermonuclear test signal within the three deepest sediment layers dated between 1953 and 1958. Within this period, USA’s first thermonuclear tests were performed at the Marshall Islands in the Pacific Ocean: Ivy Mike (10.4 Mt, Enewetak Atoll, 1952-11-01) and Operation Castle (a series of thermonuclear explosions with an overall yield of about 50 Mt, Bikini Atoll, 1954). The measured ^244^Pu/^239^Pu atom ratio in the three deepest sediment layers (1.51 ± 0.11)·10^–4^ approaches the reported ratios from samples collected at the Marshall Islands. ^244^Pu/^239^Pu ratios ranging from 2.5×10^–4^ to 5.7× 10^–4^ have been reported in sediment and soil samples from the Bikini atoll (i.e., directly impacted by the Operation Castle nuclear tests),^15^ and ^244^Pu/^239^Pu ratios measured in soil samples collected at Bikar atoll (i.e., the most northern atoll of the Republic of the Marshall Islands) are in the (2.1–3.6)·10^–4^ range.^11^ The highest ^244^Pu/^239^Pu ratio has been reported in airborne debris from Ivy Mike detonation,^10^ that is, (11.8 ± 0.7)·10^–4^. Radioactive aerosols are characterized by the stratospheric residence time between 1 and 5 years, especially when attached to fine aerosol particles (<0.02 μm diameter).^32,33^ The exchange between the stratospheric and tropospheric air masses occurs mainly during the late spring when the rising hot air and cold stratospheric air masses change place.^33^ 1952’s and 1954’s high-yield thermonuclear detonations were not the only Pu source in the stratosphere at the time. Multiple low-yield tests were performed at the Pacific and Semipalatinsk test sites introducing ^239,240,241,242^Pu isotopes mainly locally and to the troposphere. Still, some of the debris reached the stratosphere in specific detonation conditions.^33^ Therefore, the obtained ^244^Pu/^239^Pu atom ratios in this work are expected to be a mixture of Pu signatures of the earliest thermonuclear and pure fission tests. As a result, the data for close-in fallout probably differ substantially from the worldwide GF signal far from the detonation site. Likewise, the ^240^Pu/^239^Pu ratio obtained from the three deepest samples in this work (Table 2) is lower than the reported values from samples collected at the Bikini Atoll (i.e., directly impacted by the close-in fallout from the first US thermonuclear test, see Table 1). Further research is needed to confirm the different fallout patterns of ^244^Pu in the 1950s in the studied area, but this premise could be in agreement with the results observed in other studies. Cwanek et al. pointed to the early high-yielded nuclear detonations as a possible explanation for an unidentified event in the 1950s with an increase in the ^240^Pu/^239^Pu isotopic ratio from the study of the peat core in the Northern Ural (Russian Federation).^34^

### 4.3 Disentangling the Pu Signal from Studsvik

Using the proxy based on ^244^Pu, the GF input is unraveled in our sediment core, as discussed in Section 4.1. Presumably, the resulting excess of Pu should mainly originate from Studsvik releases. However, other local or regional sources must also be considered in the first instance. The fallout from the Chernobyl accident in 1986 is a source of anthropogenic radionuclides to the Baltic Sea. The critical role of this source in the Tvären bay for ^137^Cs has already been discussed in a previous study.^18^ However, the Chernobyl fallout for refractory elements such as Pu was negligible and unevenly distributed compared to that for ^137^Cs. The uneven distribution of the Chernobyl-derived ^137^Cs and Pu in the Baltic Sea sediments was shown in a previous study, where multiple cores from the Swedish coast of the Baltic Sea were analyzed.^26^ As seen in Figure 6, the trends observed for the analyzed samples do not seem to be influenced by the Chernobyl Pu fallout. Specifically, results obtained from the layer dated from 1985 to 1988 show atom ratios of 0.1527 ± 0.0020, (3.56 ± 0.15)·10^–3^, and (9.55 ± 0.45)·10^–3^ for ^240^Pu/^239^Pu, ^241^Pu/^239^Pu, and ^242^Pu/^239^Pu, respectively, and 1.295 ± 0.045 for the ^238^Pu/^239+240^Pu activity ratio. These results are incompatible with the characteristic isotopic ratios expected for the Chernobyl fallout (Table 1). Consequently, for the discussion that follows, the influence of the Chernobyl accident is considered negligible at the studied site compared to Studsvik releases. Other possible Pu inputs, such as releases from Sellafield and La Hague reprocessing plants, are expected to contribute less to the Baltic Sea, being retained mainly in the Irish Sea sediments due to the Pu particle reactive nature.^35^ The Baltic Sea exchanges around 3% of its water volume yearly, and nearly 40% of this volume enters from the North Sea through the Danish Straits.^36^ However, this input delivers insignificant amounts of Pu to the Baltic Sea compared to other local sources.^7^

**Figure 7:**
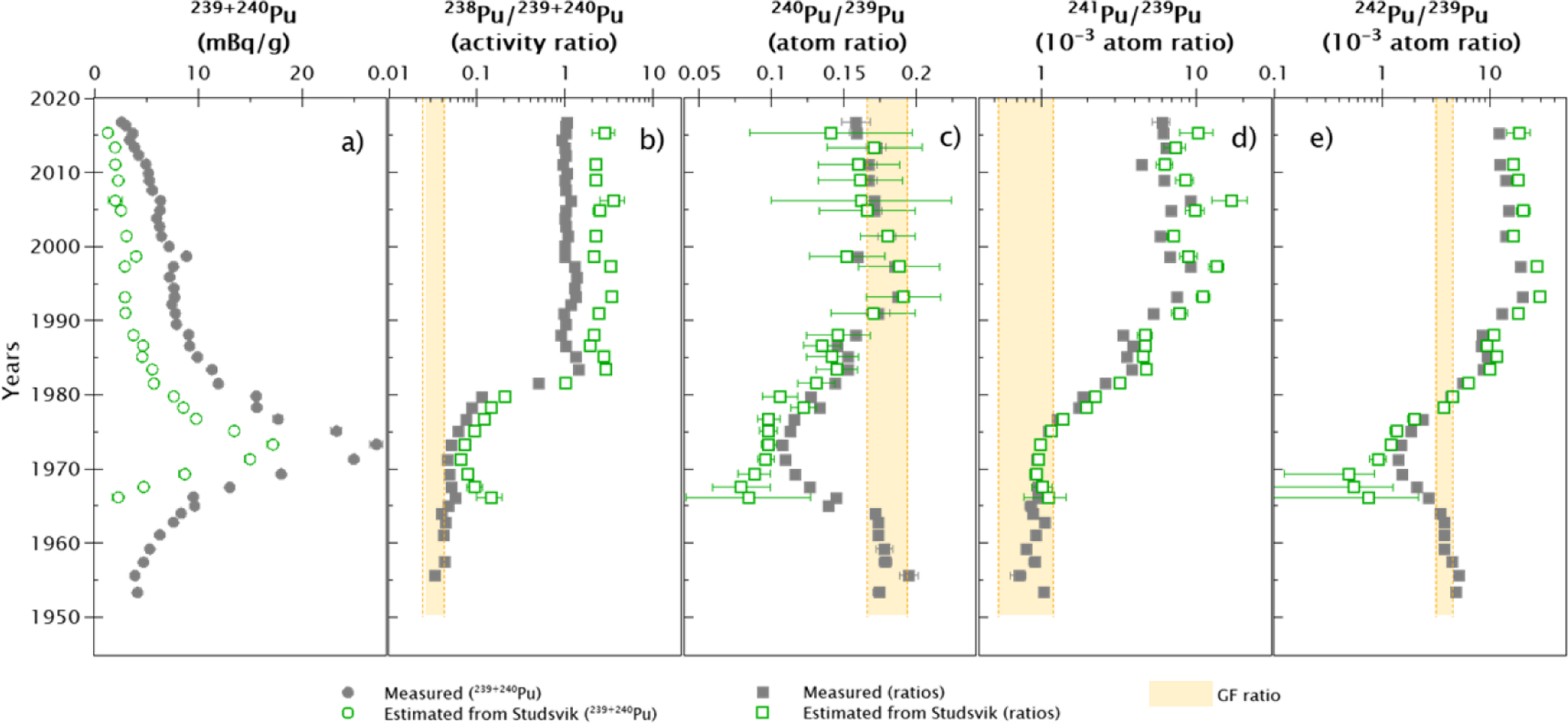
Calculations of Studsvik’s input in the sediment core. Measured ^239+240^Pu activity concentrations and Pu ratios together with the estimated contributions from Studsvik after subtracting the GF. Expected GF ratios are shaded; see Table 1 for references. The uncertainties of the date of the layers according to the ^210^Pb dating method are detailed in Figure S3.

We can assume that the influence of Studsvik aquatic discharges conditions the observed trends in the Pu isotopic ratios in core C3 (Figure 6) from the beginning of the 1960s. The trend of the Pu isotopic ratios reflects the Pu-related research activities conducted at the Studsvik nuclear facilities, which have changed and overlapped over time. Using the GF fractions obtained in Section 4.1 (see Figure 5), Studsvik releases can be revealed independently after subtracting the GF contribution. Thus, Figure 7 shows the specific contributions of Studsvik along the sediment core.

Based on our data and the historical knowledge of Studsvik operations, we can put in context the Pu discharged to Tvären by this nuclear facility. Sweden’s primary nuclear technology was based on heavy water reactors with natural uranium as fuel. After the beginning of its operation in 1959, one of the main goals of Studsvik was to gain competence in Pu separation from irradiated uranium fuel. At that time, multiple countries used civil nuclear power plants to produce weapon-grade Pu, and we can speculate that tests on such fuel were conducted at Studsvik. Indeed, the GF-corrected ^240^Pu/^239^Pu atom ratio (Figure 7) indicates that low burn-up fuel was handled at the facility around the 1970s, with ratios approaching the weapon-grade Pu quality (^240^Pu/^239^Pu atom ratio < 0.1). These ^240^Pu/^239^Pu atom ratios prevailed for several years in the radioactive effluents from Studsvik. At the beginning of the 1970s, Sweden abandoned the idea of constructing and manufacturing nuclear weapons and using heavy water reactors. The Studsvik research facility continued to perform fuel tests with conventional fuel for light water reactors. One of the international projects completed in Studsvik in the 1970s was the Studsvik Inter Ramp Project, with the primary objective of investigating failure propensity and characteristics of 20 unpressurized BWR (i.e., boiling water reactor) fuel rods.^37^ This paradigmatic political and technical change is clearly visible in the Pu atom ratio depth profiles, ^238^Pu activities, and ^238^Pu/^239+240^Pu activity ratios (Figures 2 and 7). The drastic increase in computed ^238^Pu activities with ^238^Pu/^239+240^Pu activity ratios reaching values up to 1.5 in the mid-1980s confirms this hypothesis. Additionally, we can see that ^239+240^Pu activity levels increase starting from layers dated around 1950 to reach a maximum around 1973 when the decline can be observed up to the core’s surface (Figure 3). From the mid-1970s, all Pu atom ratios referenced to ^239^Pu started to increase too, which points to Studsvik handling high burn-up fuel after the nuclear weapon program was closed. Since 1990, these ratios have been relatively constant; however, the Pu discharges have constantly decreased (Figure 7) due to improvements in the waste treatment plant at the Studsvik nuclear facility.

### 4.4 Reconstruction of Pu Historical Releases from Studsvik

**Figure 8:**
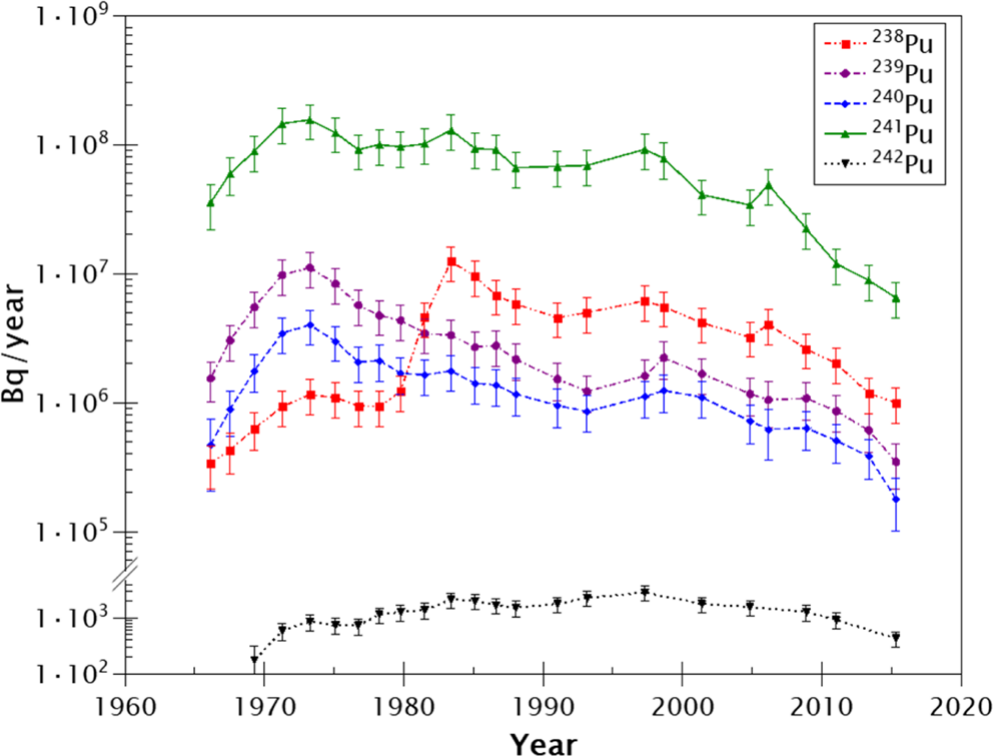
Reconstruction of Studsvik’s annual releases. Annual releases from Studsvik from this work according to the calculations described in Section 4.4. The uncertainties of the years according to the ^210^Pb dating method are detailed in Figure S3.

The earliest reported Studsvik discharges were scarce or even nonexisting for individual Pu isotopes. However, since 2002, some Pu-isotopic-specific data have been published with the annual ^239+240^Pu and ^238^Pu activities released up to 2016 (Figure S4). During this period, total discharges of 15 and 50 MBq were reported for ^239+240^Pu and ^238^Pu, respectively. It must be mentioned that the discharge data are not provided with uncertainty estimates, and in some cases, the reported ^238^Pu/^239+240^Pu activity ratios in liquid discharges were significantly higher than in the C3 sediment core as well as in the other cores collected from the vicinity of the discharge pipeline. These differences may partly be explained by uncertainties in the methodology used to monitor the liquid effluents by the facility. Only a small fraction (i.e., around 3 mL) of the discharged volume is analyzed, and homogeneity of the fluid is assumed,^18^ which may not always be valid in the case of particle reactive Pu. In addition, the determination of ^238^Pu by alpha spectrometry can be biased more easily than the determination of ^239,240^Pu due to interference of other radionuclides with similar energy to that of ^238^Pu (mainly ^228^Th, ^241^Am, and ^210^Po). We have no information if the Studsvik laboratory applies a correction for this in their calculations or if the radiochemical separation method is applied in their analysis of the discharged water. For that reason, we have only used the information of the ^239,240^Pu in our evaluation of the Pu discharge history. Based on the available data, we can calculate the site-specific transfer fraction *F*_S_ of the Pu released by Studsvik that is finally accumulated in the sediment at the sampling site. It demands comparison of the Pu inventory after subtracting the GF contribution for those layers dated from 2002 to 2016 (i.e., 51.9 ± 3.2 Bq/m^2^ for ^239+240^Pu) to the discharge data given by Studsvik for the same period. We assume that *F*_S_ has been constant during all the years Studsvik has performed aquatic discharges of Pu. Using the obtained *F*_S_ value, that is, (3.5 ± 1.0)·10^–6^/m^2^, Pu discharges from Studsvik can be estimated from all Pu isotopes as follows
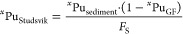
2Where ^*x*^Pu_Sediment_ is the measured Pu concentration per square meter in the sediment layer and ^*x*^Pu_GF_ is the layer-specific calculated GF fraction (with *x* = 238, 240, 241, and 242 as in eq 1, Figure 5). Thus, by using this proxy, the calculated Studsvik historical releases are shown in Figure 8. The total Pu releases since Studsvik’s commissioning (i.e., 1959) up to 2016 are (70.8 ± 5.6) MBq of ^238^Pu, (81.8 ± 6.5) MBq of ^239^Pu, (35.1 ± 2.6) MBq of ^240^Pu, (438 ± 30) MBq of ^241^Pu, and (27.3 ± 2.1) kBq of ^242^Pu equal to a total aquatic discharge of Pu of 626 MBq (Table S1). Those estimated values are about 4–7 orders of magnitude lower than the reported liquid releases by the main European Nuclear Reprocessing facilities (i.e., Sellafield and La Hague).^38,39^ Thus, Pu Studsvik releases are expected to impact locally, but due to the Baltic Sea’s semienclosed location, it should not affect the global marine environment.

## Supporting Information Available

Further information about sample preparation and measurement techniques, including a detailed description of the different groups of samples, information about the validation of AMS measurements, materials, and blanks, as well as alpha spectrometry quality control and quality assurance; results from replicate samples, Pu concentrations in the sediment core between the years 1945 to 1985, CRS dating results of core C3, and reported annual aquatic releases from the Studsvik facility; and total estimated liquid discharges from Studsvik (PDF)
