# Supplementary material for: Plutonium Signatures in a Dated Sediment Core as a Tool to Reveal Nuclear Sources in the Baltic Sea
Source: Environ Sci Technol. 2023 Jan 23;57(5):1959–69. doi: 10.1021/acs.est.2c07437 (PMC9910043; doi:10.1021/acs.est.2c07437)
Supplement: Supplementary file 1 — es2c07437_si_001.pdf [file es2c07437_si_001.pdf]

Supporting information for

Plutonium signatures in a dated sediment core as a tool to reveal nuclear sources to the Baltic Sea

Mercedes López-Lora, Grzegorz Olszewski, Elena Chamizo, Per Törnquist, Håkan Pettersson, Mats Eriksson

- **Section 1: Sample preparation and validation of the results**
- **Supporting figures**

Fig. S1 $^{240}Pu/^{239}Pu$, $^{241}Pu/^{239}Pu$ and $^{244}Pu/^{239}Pu$ from two set of samples from C3 sediment core.

Fig. S2 Pu concentrations in the sediment core C3 between the years 1945 to 1985.

Fig. S3 CRS dating results of core C3.

Fig. S4 Reported annual aquatic releases from the Studsvik facility.

- **Supporting tables**

Table S1 Total estimated liquid discharges from Studsvik (1959 – 2016).

- **References**

1. Sample preparation and validation of the results

A first set of 46 samples (Group 1) were processed at SSM laboratories (Swedish Radiation Safety Authority, Stockholm, Sweden). For this, about 3 g of sediments from all the available layers were used, and Pu fractions from those samples were purified and electrodeposited on planchets for alpha spectrometry analysis (i.e. ^{238}Pu, $^{239+240}$Pu) as it is described in methods section. ^{242}Pu was used as spike to quantify the final concentrations.

Using the information provided by alpha spectrometry (see fig. 2-a, 3-a and 3-b), 24 samples from the most relevant layers were selected to study isotopic ratios by AMS (i.e. ^{240}Pu/^{239}Pu, ^{241}Pu/^{239}Pu and ^{244}Pu/^{239}Pu). To this end, alpha planchets were leached to recover the electrodeposited Pu and, afterwards, produce the AMS cathodes[1].

Finally, 29 layers from the core were chosen to produce new samples from the remaining original material in order to complement the Pu isotopic composition by analysing the ^{242}Pu by AMS (Group 2). About 4-5 grams of samples were used and no spike was added to those samples. Therefore, only information about the isotopic composition of the samples was evaluated. To calculate the corresponding concentrations, ^{239,240}Pu activity concentrations measured by alpha spectrometry from Group 1 were used. Those samples were processed at CNA laboratories (Centro Nacional de Aceleradores, Sevilla, Spain). In addition to ^{242}Pu/^{239}Pu ratios, ^{240}Pu/^{239}Pu, ^{241}Pu/^{239}Pu and ^{244}Pu/^{239}Pu were also measured from those samples. Thus, with the study of samples from Group 2 we aimed to different goals: i) study of ^{242}Pu/^{239}Pu along the core; ii) expand the information about ^{240}Pu/^{239}Pu, ^{241}Pu/^{239}Pu and ^{244}Pu/^{239}Pu as Group 2 included layers that were not previously studied by AMS from Group 1; and iii) perform a validation of the results by analysing duplicate samples for ^{240}Pu/^{239}Pu, ^{241}Pu/^{239}Pu and ^{244}Pu/^{239}Pu.

Duplicate samples showed very consisted results for the studied ratios (Fig. S1). Although a slight tendency of higher ^{244}Pu/^{239}Pu ratios from group 1 seems to be observed, the results agree within uncertainties in the most of the cases. In all the AMS measurements, similar background levels were obtained from the instrumental blanks (i.e. below 5 total background counts in a total measurement time of 30 min for the mass 244) and the reliability of the measured ratios and the stability of the AMS analysis was control by analysing several standard samples (i.e. ColPuS[2]). Thereby, any evidence of a systematic offset during the analysis was found.

Due to the extremely low concentrations of ^{244}Pu in the samples the results were on the verge of the detection limit (i.e. 10^5 atoms), presenting high uncertainties in some cases (i.e. with a final relative uncertainty ranging from 8% to 72%) but only one sample was not possible to quantify (i.e. 1cm depth). Despite this, the reliability of those samples is supported by the good agreement between dataset from Group 1 and 2.

Final results were calculated as the weighted average considering the uncertainties in the cases of duplicate samples.

1.1 Materials and blanks

The first set of samples (Group 1) was prepared at SSM laboratories. Nitric acid (65%), hydrochloric acid (37%), ammonia (25%), ascorbic acid, hydroxylammonium hydrochloride, potassium disulfate, sodium hydroxide, sodium sulphate, thymol blue were obtained from Merck (Darmstadt, Germany). All used chemicals were of analytical grade. ^{242}Pu internal tracer was obtained from National Institute of Standards and Technology (Gaithersburg, MD, USA). Along with the samples two different blanks were processed: i) procedural blanks (i.e. processed blanks samples under the same conditions as

the samples of interest) and ii) electrodeposition blanks (i.e. spiked solutions directly electroplated together with the real samples to control the background of this specific step).

Samples from Group 2 were processed at the CNA laboratories and no spike was added to measure the ^{242}Pu in the samples. For those samples, being specifically intended for AMS and aimed for analysis of very small concentrations of ^{242}Pu and ^{244}Pu, special protocols to keep the laboratory contamination at a minimum were followed. Specifically, for the study of ^{242}Pu, the use of new materials to avoid contamination from the commonly used ^{242}Pu spike became critical. Additionally, acids of the highest purity were used in every stage, and for the AMS cathode preparation, a Fe(III) solution provided by *High Purity Standards* (HPS, England) was used, with a certified ^{238}U concentration below the 0.5 ppb level. The Nb powder added during this last step was provided by Sigma-Aldrich® (purity of 99.8%). Several procedural blanks were prepared together with those samples.

TEVA resin cartridges were obtained from Triskem International (Bruz, France). Ultra-pure water obtained from a Milli-Q system water purifier (Millipore Corp., Bedford, UK) was used for all the experiments.

Processed blanks from Group 1 and 2 presented similar backgrounds, both between them and compared with the non-processed blanks. Therefore, no additional background correction from the laboratory processing was necessary.

1.2 Alpha spectrometry quality control and quality assurance

The used plutonium separation method was controlled by using IAEA-135 marine sediment reference material. The obtained results were from 43.2±1.9 to 50.1±0.9 for ^{238}Pu, and from 216±3 to 240±3 Bq/kg for $^{239+240}$Pu. The reference confidence interval (α=0.05) for analysed Pu isotopes given in the certificate is: 41.6 to 45 Bq/kg for ^{238}Pu and 205 to 225.8 Bq/kg for $^{239+240}$Pu. The choice of IAEA-135 reference material was to ensure complete Np separation with ^{242}Pu tracer (IAEA-135 marine sediment contain alpha spectrometry measurable ^{237}Np activities).

Supporting figures

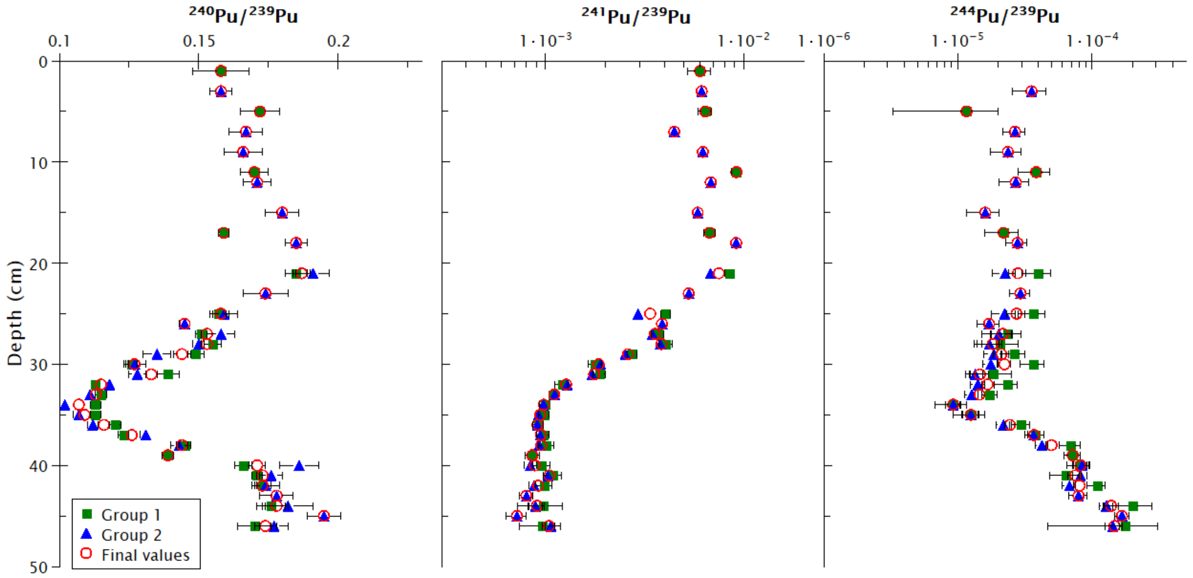

Fig. S1. $^{240}Pu/^{239}Pu$, $^{241}Pu/^{239}Pu$ and $^{244}Pu/^{239}Pu$ from two set of samples from C3 sediment core. Pu atom ratios from Group 1 and 2 (see section 1 from Supplemental information). The final values are calculated from the weighted average for replicate samples.

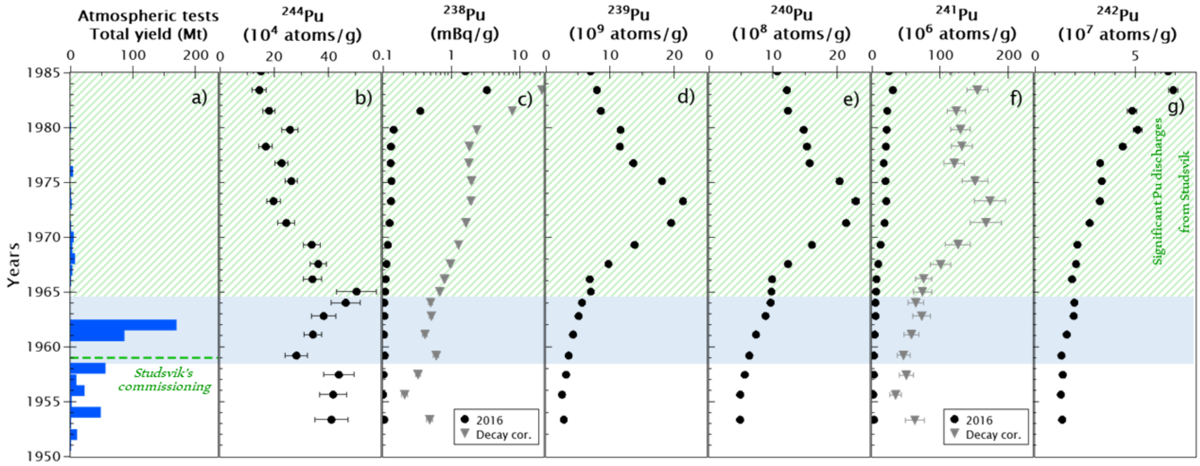

Fig. S2. Pu concentrations in the sediment core C3 between the years 1945 to 1985. Pu concentrations from all the isotopes studied in this work from layers dated up to 1985. ^{238}Pu (c) and ^{241}Pu (f) concentrations are decay corrected to 2016 (black dots) and to the date of the layer according to the ^{210}Pb dating method (grey triangles). The left plot (a) represents the total yield from atmospheric nuclear test (1945 – 1980, [3]) and the green dashed line indicates the commission year of Studsvik nuclear facility. The green dashed area represents the layers of the sediment core significantly affected by the Pu discharges from Studsvik (section 4.1). The uncertainties of the date of the layers according to the ^{210}Pb dating method are detail in Fig. S3.

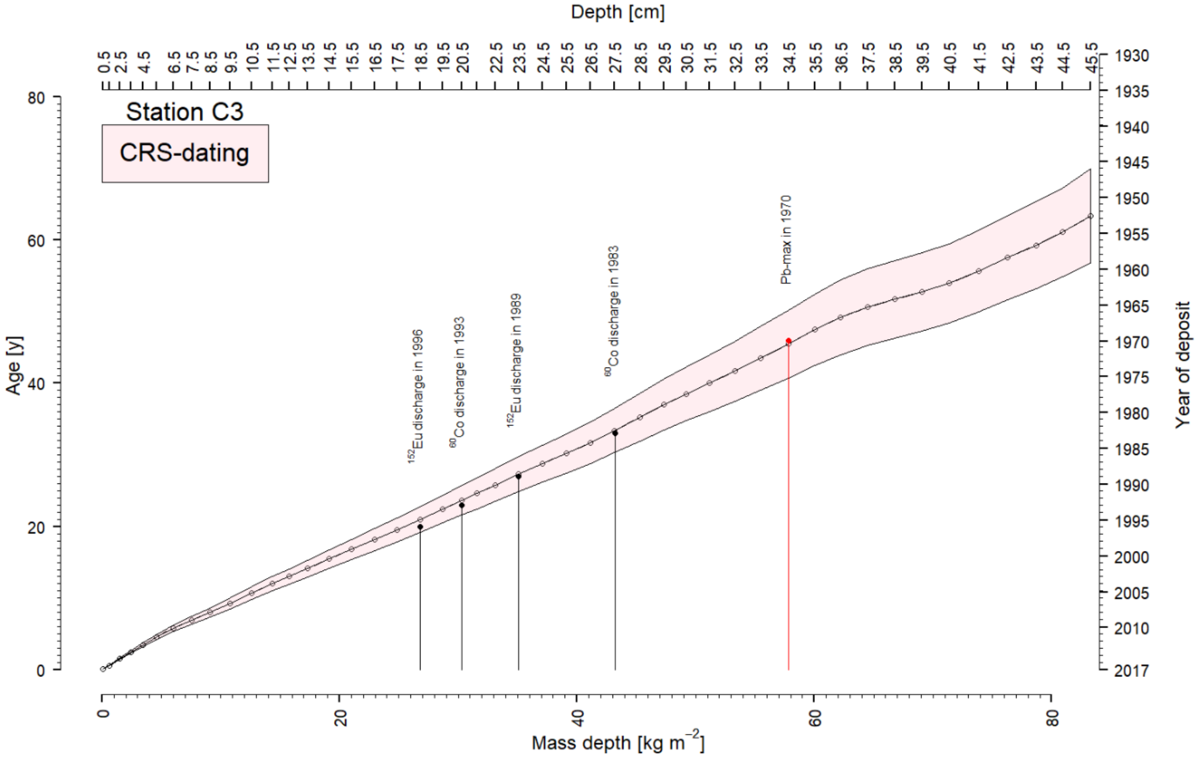

Fig. S3 CRS dating results of core C3. The graph shows the age/year of deposit of the sediment slide, solid line shows the mean age and the coloured area show one standard deviation of the results. The statistics are based on a Monte Carlo simulation running 1000 dating simulations. The CRS dating (Constant Rate of Supply) is validated by using stable lead and additional time markers according to the Studsvik discharge history records and the observed activity concentrations in the sediment core. [4]

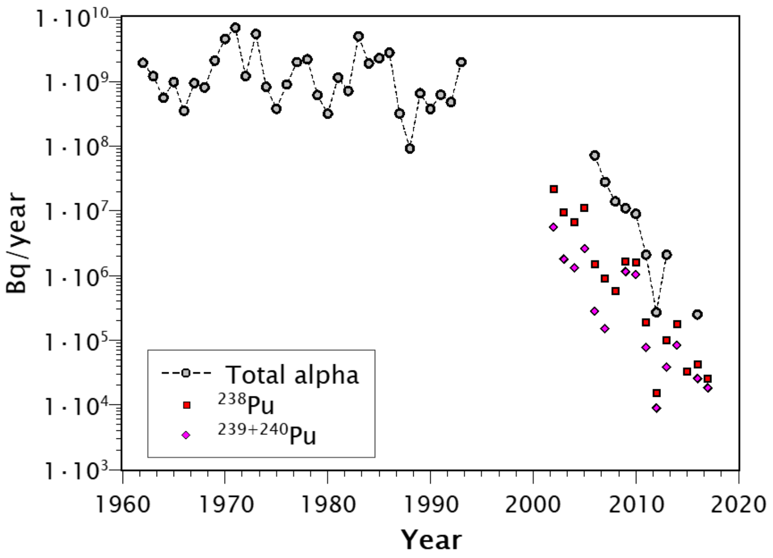

Fig. S4 Reported annual aquatic releases from the Studsvik facility. Data provided by Studsvik.

Supporting tables

Table S1 Total estimated liquid discharges from Studsvik (1959 – 2016).

	^{238}Pu	^{239}Pu	^{240}Pu	^{241}Pu	^{242}Pu
Atoms	$(2.83 \pm 0.22)\cdot10^{16}$	$(8.97 \pm 0.71)\cdot10^{19}$	$(1.047 \pm 0.079)\cdot10^{19}$	$(2.86 \pm 0.19)\cdot10^{17}$	$(4.65 \pm 0.36)\cdot10^{17}$
mg	0.112 ± 0.010	35.6 ± 2.9	4.17 ± 0.31	0.1149 ± 0.0077	0.187 ± 0.014
MBq	70.8 ± 5.6	81.8 ± 6.5	35.1 ± 2.6	438 ± 30	0.0273 ± 0.0021

References

(1) Chamizo, E.; Rääf, C.; López-Lora, M.; García-Tenorio, R.; Holm, E.; Rabesiranana, N.; Pédehontaa-Hiaa, G. Insights into the Pu Isotopic Composition (239Pu, 240Pu, and 241Pu) and 236U in Marshland Samples from Madagascar. *Science of The Total Environment* **2020**, *740*, 139993. https://doi.org/10.1016/j.scitotenv.2020.139993.

(2) Dittmann, B.-A.; Buompane, R.; Chamizo, E.; Christl, M.; Dewald, A.; Dunai, T.; Feuerstein, C.; Fifield, K.; Fröhlich, M.; Heinze, S.; Marzaioli, F.; Münker, C.; Petraglia, A.; Sirignano, C.; Strub, E.; Synal, H.-A.; Terrasi, F.; Tims, S.; Wallner, A. ColPuS, a New Multi-Isotope Plutonium Standard for Accelerator Mass Spectrometry. *Nucl Instrum Methods Phys Res B* **2019**, *438*, 189–192. https://doi.org/10.1016/j.nimb.2018.04.032.

(3) UNSCEAR. Annex C: Exposures from Man-Made Sources of Radiation. In *Sources and effect of ionizing radiation. UNSCEAR 2000 Report Vol. I*; 2000.

(4) Törnquist, P.; Eriksson, M.; Olszewski, G.; Carlsson, M.; López-Lora, M.; Pettersson, H. On the Use of Dated Sediments to Investigate Historical Nuclear Discharges. *Marine Pollution Bulletin (under review)* **2022**.
